# Perceived protective behavioral changes in Chinese residents post-dynamic zero-COVID policy lifting: a cross-sectional study

**DOI:** 10.3389/fpubh.2024.1439749

**Published:** 2024-10-30

**Authors:** Yuan-Yuan Song, Ling Xu, Dan Liu, Mei Feng, Cui Yang, Yan Jiang, Ying Wu

**Affiliations:** ^1^Department of Critical Care Medicine, West China Hospital, Sichuan University/West China School of Nursing, Sichuan University, Chengdu, Sichuan, China; ^2^Department of Respiratory and Critical Care Medicine, West China Hospital, Sichuan University/West China School of Nursing, Sichuan University, Chengdu, Sichuan, China; ^3^Department of Respiratory and Critical Care Medicine, West China Hospital, Sichuan University, Chengdu, Sichuan, China; ^4^Department of Nursing, West China Hospital, Sichuan University/Evidence-based Nursing Center, West China Hospital, Sichuan University, Chengdu, Sichuan, China

**Keywords:** COVID-19, public policy, perceived behavioral change, coping styles, negative emotions, risk perception, protective behaviors

## Abstract

**Objective:**

To investigate how Chinese residents perceived changes in their protective behaviors in the early stage after the lifting of the dynamic zero-COVID policy, and to explore the associations between the overall perceived change and factors such as demographic and health-related information, COVID-19 related perceptions, negative emotions, and coping styles.

**Methods:**

This cross-sectional study involved 798 Chinese residents who completed an online questionnaire between 16 and 25 December 2022. The questionnaire covered demographic and health-related information, COVID-19 related perceptions, negative emotions, coping styles, and perceived changes in protective behaviors. Multiple linear stepwise regression analysis was used to determine the factors associated with the overall perceived change in protective behaviors.

**Results:**

The mean score for perceived protective behavioral change among participants was 61.38 (SD = 10.20), which was significantly higher than the hypothesized no-change value of 49 (*p* < 0.001). The mean scores for each of the 15 behaviors (excluding the two vaccination-related items) were significantly greater than the hypothesized no-change value of 3 (*p* < 0.001). The mean scores for the two vaccination-related items were significantly greater than the hypothesized no-change value of 2 (*p* < 0.001). Among all behaviors, avoiding dining out or gathering with friends had the highest mean score (Mean = 4.16), while engaging in regular physical activity had the lowest (Mean = 3.32). Avoiding dining out or gathering with friends had the highest percentage of individuals reporting an increase (71.3%), whereas maintaining a social distance of more than 1 m had the highest percentage of individuals reporting a decrease (17.5%). Regression analysis indicated that age, worry, positive coping, female sex, negative coping, and perceived severity were associated with the overall perceived change in protective behaviors, with worry being the most predictive variable.

**Conclusion:**

This study suggested that Chinese residents perceived an increase in their protective behaviors in the early stage after the policy change, with varying magnitudes across behaviors. We identified some potentially modifiable factors associated with perceived protective behavioral change, with worry emerging as the strongest predictor, followed by positive coping, negative coping, and perceived severity. These insights offer valuable information for developing effective communication strategies, psychological support, and comprehensive models in health behavior research.

## Introduction

The World Health Organization (WHO) declared the COVID-19 outbreak a pandemic on March 11, 2020 (1). To prevent COVID-19 transmission and control the epidemic, governments worldwide implemented strict public health interventions aimed at altering social and health behaviors, such as lockdowns, quarantine, travel restrictions, mask wearing, specific hygiene measures, contact tracing, nucleic acid testing, and social distancing (2–4). The Chinese government introduced particularly stringent measures under the dynamic zero-COVID policy in April 2020 (5). On December 7, 2022, China announced “the 10-point measures” to further optimize COVID-19 prevention and control measures (5), marking the end of its most restrictive guidelines and signaling a shift from government-led imposed regulations to voluntary protective behaviors.

Throughout the pandemic, behavioral change has been central to mitigating the spread of COVID-19, with the stringency of policies significantly influencing public protective behaviors across countries (6, 7). Knell et al. (8) found that adults reported a decrease in physical activity, while sleep patterns and negative health behaviors remained unchanged during the “Stay-at-Home” orders. Some studies observed declines in positive behaviors such as physical activity and sleep, while unhealthy behaviors like poor diet and smoking increased during the COVID-19 pandemic lockdowns and social distancing measures (9–13). Gibson et al. (14) noted an increase in intentions to practice social distancing, but a decrease in actual social distancing behaviors over time in the United States. Li et al. (15) found that changes in perceived risk over time resulted in corresponding adjustments in protective behaviors. Dryhurst et al. (16) highlighted significant cultural and regional differences in protective behaviors. Unlike many other countries that lifted official regulations early, China maintained its dynamic zero-COVID policy for almost 3 years. This study focuses on exploring how Chinese individuals adjusted their protective behaviors after this policy was lifted. Individuals exhibit different levels of engagement with COVID-19 public health behaviors based on their various characteristics. The literature showed that in the United States, vaccine acceptance was low among pregnant or breastfeeding women (17), but higher among men, individuals with higher education, those over 45 years old, and people with higher incomes (18). A rapid review suggested that adherence to COVID-19 guidelines was more common among women, older individuals, those who trust governments, and those who perceive COVID-19 as threatening (19). Pedersen and Favero (20) concluded that attitudes and beliefs about COVID-19 were primary drivers of social distancing behavior. The Health Belief Model (HBM) emphasizes that individuals are more likely to engage in protective behaviors if they perceive a high risk of a health condition (21). The Protective Action Decision Model (PADM) focuses on how immediate threat perceptions influence protective actions during crises (22). Recent applications of these theories have demonstrated their relevance in understanding contemporary health behaviors and indicate the associations between risk perceptions and protective behaviors (23, 24). Negative emotions such as fear and worry have been found the motivators for COVID-19 protective behaviors (25, 26). Additionally, people have adopted various coping strategies in response to the pandemic, which are crucial for mental well-being (27, 28) and may impact adherence to protective behaviors, particularly in the absence of prior experience (29). Lazarus and Folkman’s Stress and Coping Theory suggests that stress arises from how individuals appraise a situation and their perceived ability to cope with it (30). Recent studies have continued to explore and validate this framework in contemporary settings (31, 32), emphasizing the role of coping mechanisms in shaping behaviral responses to stress.

Given the difficulty in accurately measuring actual behavioral changes over a short period, our study focuses on perceived changes in protective behaviors. This approach involves capturing individuals’ subjective assessments of their own behavior changes following the policy shift, providing a practical and informative perspective on how individuals respond to evolving public health interventions. Perceived behavioral changes offer valuable insights into how individuals interpret and adjust their behaviors in response to policy alterations, which is crucial for understanding public attitudes and guiding health interventions. Thus, we aimed to understand how Chinese residents perceived changes in their protective behaviors in the early stage following the lifting of the dynamic zero-COVID policy. We also sought to explore the associations between the overall perceived change and factors such as demographic and health-related information, COVID-19 related perceptions, negative emotions, and coping styles. Based on existing literature and related theories, such as HBM, PADM, and Lazarus and Folkman’s Stress and Coping Theory, we propose the following hypotheses:

1. There will be significant perceived changes in protective behaviors among Chinese residents in the early stage following the lifting of the dynamic zero-COVID policy.
2. Higher risk perception, negative emotions, and positive coping are expected to be associated with increased perceived protective behaviors, while negative coping will be negatively associated with the overall perceived change in protective behaviors.

This knowledge is essential for understanding the public’s response to evolving health guidelines and for shaping future strategies to ensure public safety and preparedness as health measures transition.

## Methods

### Study design and sample

The present study used an online questionnaire-based cross-sectional design with a mixed-method sampling approach. Initially, convenience sampling was employed to reach individuals within the researcher’s direct contacts. Subsequently, snowball sampling was used to expand the sample size by encouraging participants to refer individuals outside of their immediate circles. This approach aimed to increase diversity by indirectly reaching participants from various demographic backgrounds. This study targeted individuals who were aged 18 years or older, able to read and understand Chinese, and had access to the internet. A total of 843 Chinese individuals visited the online survey and provided informed consent. Data cleaning processes were then conducted, including the removal of records with duplicated data (*n* = 42) and logistic errors (*n* = 3), resulting in 798 unique records for data analysis (valid response rate: 94.7%). A flowchart detailing these steps has been included to visually outline the data cleaning process (Supplementary Figure S1).

### Questionnaires

#### Demographic and health-related information

This section included information on variables such as sex, age, educational level, relationship status, living arrangements, and place of residence.

#### COVID-19 related perceptions

This section included perceived susceptibility to COVID-19, perceived severity, and perceived impact of COVID-19, all of which were designed based on previous research (33).

Perceived susceptibility to COVID-19 included 2 items that measured the likelihood of contracting COVID-19 for oneself and one’s family members. A five-point Likert scale (1 = very little to 5 = very much) was used, and higher scores suggested higher levels of perceived susceptibility. The Cronbach’s alpha coefficient for this measure in this study was 0.883.

Perceived severity of COVID-19 was measured by a single item that asked about the individual’s perception of the seriousness of COVID-19 infection. A five-point Likert scale was used (1 = not serious to 5 = very serious), with higher scores reflecting greater perceived severity.

Perceived impact of COVID-19 consisted of 4 items. Participants were asked to rate whether COVID-19 had affected any part of their daily lives (impact on work/studies, finances, family relationships, and social contact). The items were rated on a five-point Likert scale (1 = very little to 5 = very much), with higher scores indicating greater perceived impact. In this study, the Cronbach’s alpha coefficient for this measure was 0.785.

#### Negative emotions

This section included fear and worry, which were designed based on previous research (33).

Fear was assessed by a single item. Participants were asked to rate their level of fear of COVID-19 on a five-point Likert scale (1 = very little to 5 = very much), with higher scores indicating greater levels of fear.

Worry was assessed by 8 items. Participants were asked to rate their level of worry regarding various aspects related to COVID-19 (contracting COVID-19, family members or friends contracting COVID-19, transmitting COVID-19 to others, physical symptoms, sequelae, financial burden, and stigmatization due to the infection, and reinfection). The items were rated on a five-point Likert scale (1 = very little to 5 = very much), with higher scores indicating greater levels of worry. The Cronbach’s alpha coefficient for worry in this study was 0.928.

#### Coping style

The Trait Coping Style Questionnaire (TCSQ) is a 20-item instrument designed to measure the relatively stable coping style of individuals with certain personality tendencies to different events in life (34). The TCSQ includes two domains: positive coping (PC) and negative coping (NC). Each dimension consists of 10 items rated on a five-point Likert scale (1 = absolutely not to 5 = absolutely yes). The total score for each subscales ranges from 10 to 50, and higher scores indicate a stronger tendency toward the corresponding coping style. The scale has been found to be valid and reliable in the Chinese population (35, 36). In the current study, the Cronbach’s alpha coefficient was 0.856 for PC and 0.872 for NC.

#### Perceived changes in protective behaviors

This section was designed based on previous studies (37–39) to gather information on participants’ perceived changes in protective behaviors since the release of “the 10-point measures.” A total of 17 items were developed through a review of existing literature and refined through internal discussions to ensure consistency and reliability. These items covered various protective behaviors, such as avoiding going to public places with large crowds, maintaining a social distance of more than 1 m, and vaccination-related behaviors. With the exception of two vaccination-related items, which were rated on a three-point scale (1 = not vaccinated and refuse to vaccinate, 2 = vaccinated, and 3 = not vaccinated but plan to vaccinate), the remaining items were scored on a five-point scale (1 = much less than before, 2 = slightly less than before, 3 = as often as before, 4 = slightly more than before, and 5 = much more than before). All items were summed to create one composite score ranging from 17 to 81, with a score of 49 representing no change in behaviors. Higher scores reflected greater increases in self-reported protective behaviors. We also created count variables to indicate the number of participants who reported an increase, no change, or decrease in each behavior. Furthermore, participants were asked whether they had purchased items such as antiviral drugs, masks, home oxygen generators, oxygen saturation monitors, disinfectants, antigen test kits, and other items. The Cronbach’s alpha coefficient for this section was 0.889.

A table summarizing the details of the questionnaires for all variables is provided in Supplementary Table S1.

### Data collection procedure

The study was approved by the Ethics Committee of the first author’s affiliation (Approval No. 2022.1943). Data were collected through online surveys using the Wenjuanxing website^1^ from December 16, to December 23, 2022. The questionnaire link was distributed through WeChat, the most widely used social media platform in China. The participants first encountered an informed consent form detailing an introduction to the study, the voluntary, anonymous and confidential nature of participation, guides for completing the questionnaire, an invitation to participate, and an informed consent option. All participants were asked to select “agree to participate” before proceeding with the rest of the questionnaire. The research team used IP address restriction technology, thus, users with the same IP address could complete the survey only once. The questionnaire took approximately 10 min to complete. Data collection was securely managed on the Wenjuanxing platform, ensuring compliance with data protection regulations. In addition, we implemented strict quality control measures, including conducting pre-surveys to test the survey instrument and thoroughly cleaning and validating the data.

### Data analysis

All statistical analyses were performed with SPSS Version 22 (IBM Corporation, New York, United States). Descriptive statistics were used to present the findings. Single-sample *t*-tests were used to assess whether the change scores for individual protective behaviors, as well as the overall change score, were statistically significant compared to the hypothesized no-change value. Differences between groups were evaluated using the independent samples *t*-test for comparisons between two groups or one-way analysis of variance (ANOVA) for comparisons among multiple groups. Pearson’s correlation analyses were carried out to test the relationships between COVID-19 related perceptions, coping styles, and the overall perceived change in protective behaviors. Multiple linear stepwise regression analysis was conducted to examine the factors associated with the overall perceived change in protective behaviors. Statistically significant variables (*p* < 0.05) in univariate analysis and variables deemed professionally significant were included in the model. Only variables with a two-sided *p-*value of less than 0.05 were retained in the final model.

## Results

### Participant characteristics

As shown in Table 1, most participants were female (76.2%), aged 31–45 years (42.5%), had an undergraduate or associated degree (76.8%), were married or in a relationship (76.2%), lived with others (80.2%), resided in urban areas (84.7%), had a high employment-related risk of contracting COVID-19 (70.7%), and were either healthcare providers themselves or had a family member who was a health care provider (63.5%). Additionally, 72.3% of participants reported having no chronic diseases. A total of 12.8% of all participants lived with or cared for infants or toddlers, 21.3% lived with or cared for 4–6 years old children, 30.5% lived with or cared for older adult individuals (over 65 years old), and 17.4% lived with or cared for people with chronic conditions. Only 17.3% rated their family’s economic status as good or very good. A total of 48.2% rated their physical health as good or very good, while 62.4% rated their mental health as good or very good. Regarding COVID-19 infection history, 30.6% reported having been infected, and 40.7% reported that their family members or friends had been infected. A total of 64.4% reported having current influenza-like symptoms, and 63.4% reported that their family members or other housemates were experiencing similar symptoms.

**Table 1:** Comparison of perceived protective behavioral change among different sample groups (*N* = 798).

Group variable	*n* (%)	Perceived protective behavioral change Mean (SD)	*t/F*	*p*
Sex
Male	190(23.8)	57.95(10.53)	−5.414	**<0.001**
Female	608(76.2)	62.46(9.86)		
Age, years
≤30	312(39.1)	59.76(10.36)	8.307	**<0.001**
31–45	339(42.5)	61.87(10.33)		
≥46	147(18.4)	63.71(9.00)		
Educational level
High school and below	122(15.3)	61.00(11.67)	0.762	0.467
Undergraduate or associate degree	613(76.8)	61.60(9.79)		
Graduate	63(7.9)	60.05(11.11)		
Relationship status
Married or in a relationship	608(76.2)	62.18(9.61)	3.597	**<0.001**
Single	190(23.8)	58.85(11.58)		
Living arrangements
Living alone	158(19.8)	58.77(10.92)	−3.631	**<0.001**
Living with others	640(80.2)	62.03(9.92)		
Place of residence				
Urban	676(84.7)	61.23(10.17)	−0.994	0.321
Rural	122(15.3)	62.23(10.36)		
Employment-related risk of contracting COVID-19				
Yes	564(70.7)	61.90(9.86)	2.201	**0.028**
No	234(29.3)	60.15(10.91)		
Whether oneself or one’s family member was a health care provider				
Yes	507(63.5)	62.09(9.75)	2.590	**0.010**
No	291(36.5)	60.15(10.85)		
Chronic diseases				
Yes	221(27.7)	62.00(10.06)	1.062	0.288
No	577(72.3)	61.15(10.25)		
Caregiving or cohabitation with infants or toddlers				
Yes	102(12.8)	63.36(8.66)	2.404	**0.017**
No	696(87.2)	61.09(10.38)		
Caregiving or cohabitation with 4–6 years old children				
Yes	170(21.3)	62.58(9.44)	1.728	0.084
No	628(78.7)	61.06(10.38)		
Caregiving or cohabitation with older adult individuals (>65 years old)				
Yes	243(30.5)	62.91(9.80)	2.806	**0.005**
No	555(69.5)	60.72(10.31)		
Caregiving or cohabitation with people with chronic diseases				
Yes	139(17.4)	63.00(9.60)	2.058	**0.040**
No	659(82.6)	61.04(10.30)		
Perceived family economic status				
Very poor or poor or moderate	660(82.7)	61.39(10.09)	0.056	0.955
Very good or good	138(17.3)	61.34(10.77)		
Perceived current physical health status				
Very poor or poor or moderate	413(51.8)	61.81(10.12)	1.230	0.219
Very good or good	385(48.2)	60.92(10.29)		
Perceived current mental health status				
Very poor or poor or moderate	299(37.5)	61.34(10.31)	−0.086	0.931
Very good or good	499(62.5)	61.41(10.15)		
History of COVID-19 infection				
Yes	244(30.6)	61.45(9.90)	1.692	0.185
Have no idea	97(12.2)	59.63(11.04)		
No	457(57.3)	61.72(10.16)		
History of COVID-19 infection among family members or friends				
Yes	325(40.7)	61.89(10.09)	0.937	0.392
Have no idea	89(11.2)	60.33(11.24)		
No	384(48.1)	61.20(10.05)		
Current influenza-like symptoms in self				
Yes	514(64.4)	61.58(9.91)	0.719	0.472
No	284(35.6)	61.04(10.72)		
Current influenza-like symptoms in family members or other housemates				
Yes	506(63.4)	61.68(9.86)	1.061	0.289
No	292(36.6)	60.88(10.77)		

SD, standard deviation. Significant p-values (p < 0.05) are bolded.

### Perceived changes in protective behaviors

**Figure 1:**
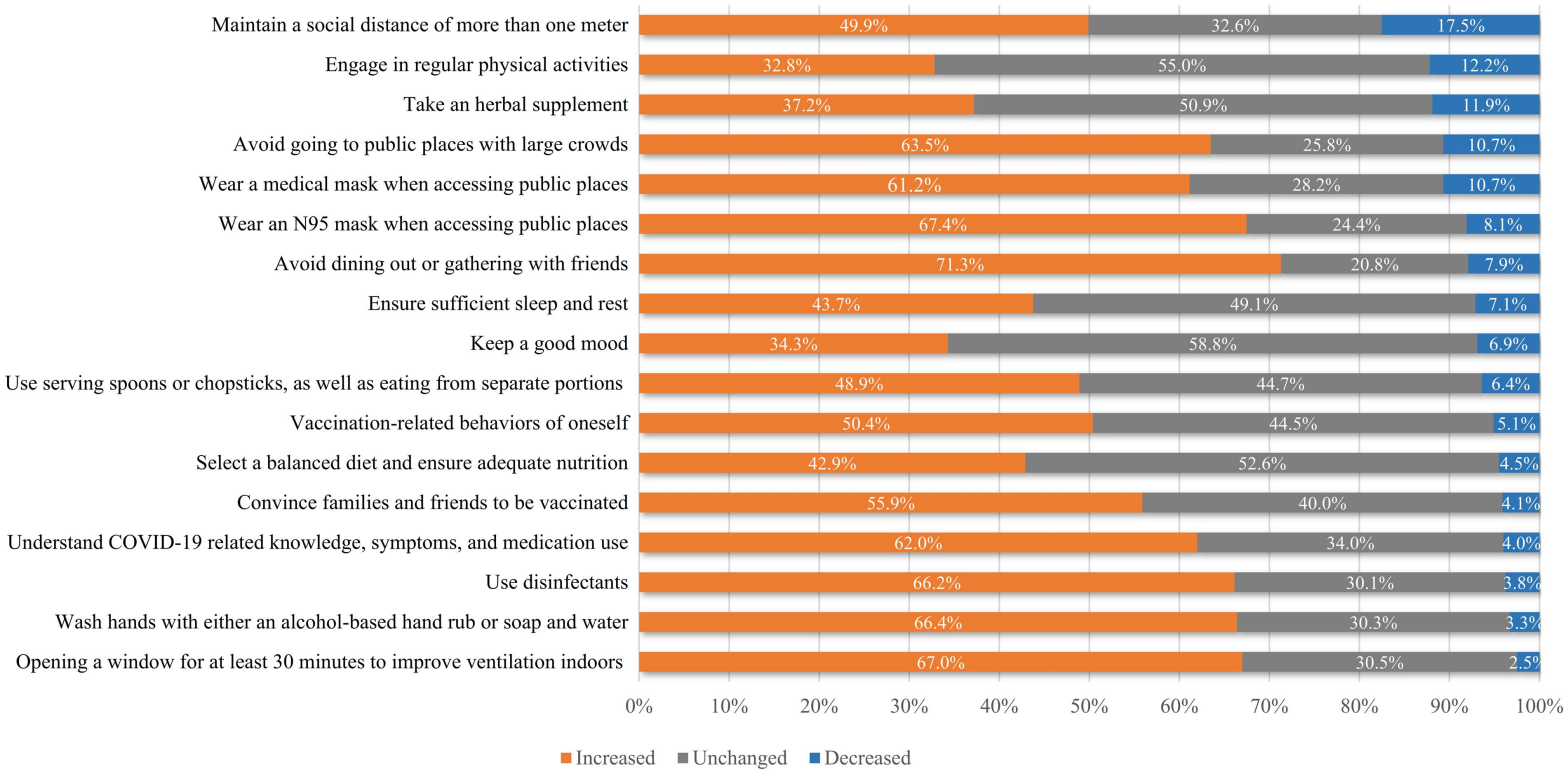
Perceived changes in protective behaviors after the lifting of the dynamic zero-COVID policy.

**Figure 2:**
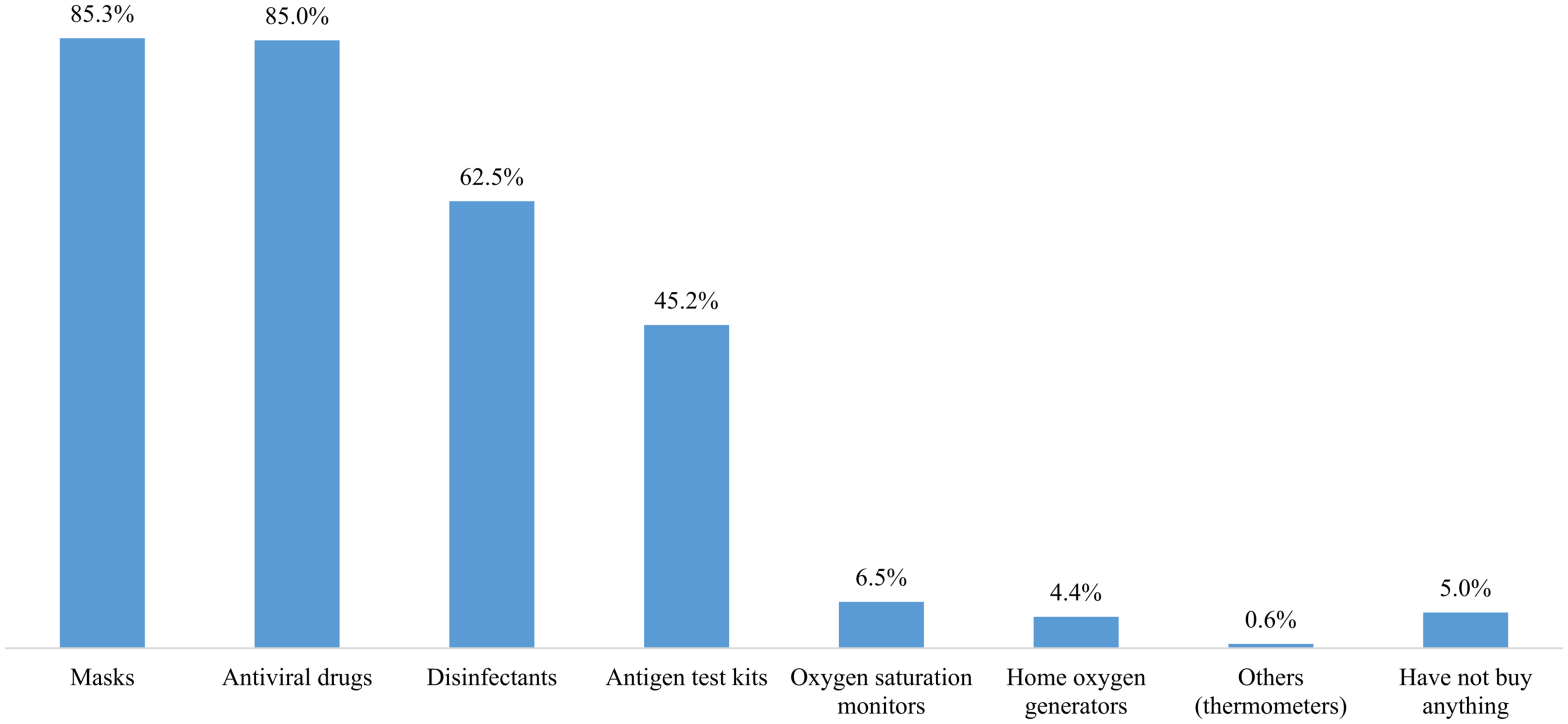
Commonly purchased medical products after the lifting of the dynamic zero-COVID policy.

The details of the participants’ perceived changes in protective behaviors are shown in Table 2 and Figure 1. The total scores for perceived protective behavioral change among the participants ranged from 25 to 81, with a mean score of 61.38 (SD = 10.20), which was significantly higher than the hypothesized no-change value of 49 (*p* < 0.001). The mean scores for each of the 15 behaviors (excluding the two vaccination-related items) were significantly greater than the hypothesized no-change value of 3 (*p* < 0.001). For the two vaccination-related items, the mean scores were significantly greater than the hypothesized no-change value of 2 (*p* < 0.001). The three items with the highest mean scores were avoiding dining out or gathering with friends (Mean = 4.16), opening a window for at least 30 min to improve ventilation indoors (Mean = 4.08), and washing hands with either an alcohol-based hand rub or soap and water (Mean = 4.06). Conversely, the three items with the lowest mean scores were keeping a good mood (Mean = 3.40), taking herbal medicines or supplements (Mean = 3.35), and engaging in regular physical activity (Mean = 3.32). The behavior with the highest percentage of individuals reporting an increase was avoiding dining out or gathering with friends (71.3%). In contrast, maintaining a social distance of more than 1 m had the highest percentage of individuals reporting a decrease (17.5%). During this period, the most commonly purchased medical products were masks (85.3%), antiviral drugs (85.0%), disinfectants (62.5%), and antigen test kits (45.2%) (Figure 2).

**Table 2:** Perceived changes in protective behaviors among participants (*N* = 798).

Variable	Score range	Min	Max	Mean (SD)	The hypothesized no-change value	*t*	*p*
The overall perceived change in protective behaviors	17–81	25	81	61.38(10.20)	49	34.293	<0.001
Avoid dining out or gathering with friends	1–5	1	5	4.16(1.07)	3	30.576	<0.001
Opening a window for at least 30 min to improve ventilation indoors	1–5	1	5	4.08(0.96)	3	31.809	<0.001
Wash hands with either an alcohol-based hand rub or soap and water	1–5	1	5	4.06(0.99)	3	30.195	<0.001
Use disinfectants	1–5	1	5	4.03(0.99)	3	29.577	<0.001
Wear an N95 mask when accessing public places	1–5	1	5	3.98(1.13)	3	24.469	<0.001
Avoid going to public places with large crowds	1–5	1	5	3.97(1.12)	3	24.507	<0.001
Wear a medical mask when accessing public places	1–5	1	5	3.92(1.22)	3	21.327	<0.001
Understand COVID-19 related knowledge, symptoms, and medication use	1–5	1	5	3.87(0.95)	3	25.890	<0.001
Use serving spoons or chopsticks, as well as eating from separate portions rather than from the same plates	1–5	1	5	3.67(1.04)	3	18.174	<0.001
Select a balanced diet and ensure adequate nutrition	1–5	1	5	3.58(0.92)	3	17.670	<0.001
Ensure sufficient sleep and rest	1–5	1	5	3.54(0.96)	3	15.832	<0.001
Maintain a social distance of more than 1 m	1–5	1	5	3.51(1.25)	3	11.534	<0.001
Keep a good mood	1–5	1	5	3.40(0.92)	3	12.211	<0.001
Take herbal medicines or supplements	1–5	1	5	3.35(1.08)	3	9.134	<0.001
Engage in regular physical activities	1–5	1	5	3.32(1.01)	3	8.902	<0.001
Convince families and friends to be vaccinated	1–3	1	3	2.52(0.58)	2	25.342	<0.001
Vaccination-related behaviors of oneself	1–3	1	3	2.45(0.59)	2	21.572	<0.001

SD, standard deviation. All *p*-values are < 0.05.

### Comparison of perceived protective behavioral change among different sample groups

There were significant differences in perceived protective behavioral change by sex, age group, relationship status, living arrangements, employment-related risk of contracting COVID-19, whether participants or their family members healthcare providers, and whether they lived with or cared for infants or toddlers, older adult individuals (over 65 years old), or people with chronic conditions. Details of these differences are presented in Table 1.

### Correlations between COVID-19 related perceptions, negative emotions, coping styles, and perceived protective behavioral change

Perceived susceptibility to COVID-19 (*r* = 0.152, *p* < 0.001, df = 796), perceived severity (*r* = 0.259, *p* < 0.001, df = 796), perceived impact of COVID-19 (*r* = 0.163, *p* < 0.001, df = 796), fear (*r* = 0.242, *p* < 0.001, df = 796), worry (*r* = 0.329, *p* < 0.001, df = 796), and positive coping (*r* = 0.228, *p* < 0.001, df = 796) were significantly correlated with perceived protective behavioral change (*p* < 0.05). Among these, worry exhibited the strongest correlation with perceived protective behavioral change, as shown in Table 3.

**Table 3:** Correlations between COVID-19 related perceptions, coping styles, and perceived protective behavioral change among participants (*N* = 798).

Variable	Min	Max	Mean (SD)	*r (p)*
Perceived susceptibility	2	10	8.20(1.87)	0.152 (**<0.001**)
Likelihood of contracting COVID-19	1	5	4.15(0.99)	0.140 (**<0.001**)
Likelihood of one’s family members contracting COVID-19	1	5	4.05(0.98)	0.149 (**<0.001**)
Perceived severity	1	5	3.59(0.89)	0.259 (**<0.001**)
Perceived impact	4	20	13.73(3.44)	0.163 (**<0.001**)
Impact of COVID-19 on work or study	1	5	3.80(0.92)	0.156 (**<0.001**)
Impact of COVID-19 on social contacts	1	5	3.59(1.14)	0.110 (**0.002**)
Impact of COVID-19 on finances	1	5	3.57(1.08)	0.149 (**<0.001**)
Impact of COVID-19 on family relationships	1	5	2.78(1.25)	0.104 (**0.003**)
Fear	1	5	3.34(0.98)	0.242 (**<0.001**)
Worry	8	40	29.43(6.97)	0.329 (**<0.001**)
Worried about family members or friends contracting COVID-19	1	5	4.05(0.94)	0.302 (**<0.001**)
Worried about transmitting COVID-19 to others	1	5	3.95(0.97)	0.335 (**<0.001**)
Worried about reinfection	1	5	3.90(1.04)	0.303 (**<0.001**)
Worried about COVID-19 related physical symptoms	1	5	3.81(1.02)	0.276 (**<0.001**)
Worried about COVID-19 related sequela	1	5	3.73(1.08)	0.277 (**<0.001**)
Worried about COVID-19 related financial burden	1	5	3.58(1.14)	0.221 (**<0.001**)
Worried about contracting COVID-19	1	5	3.50(1.07)	0.257 (**<0.001**)
Worried about stigmatization due to the infection	1	5	2.91(1.25)	0.210 (**<0.001**)
Coping styles				
Positive coping	10	50	34.14(5.57)	0.228 (**<0.001**)
Negative coping	10	50	29.13(6.59)	0.021 (0.557)

SD, standard deviation. Significant *p*-values (*p* < 0.05) are bolded.

### Multiple linear regression model

We included variables that were statistically significant in the preceding univariate analyses and those considered theoretically relevant in the multivariate analysis. The normality probability plot and scatterplots of residuals showed that the data met the basic assumptions of linear regression analysis for normality, linearity, and homoscedasticity. Variance inflation factors (VIFs) ranged from 1.032 to 1.522, suggesting the absence of serious multicollinearity. The model showed that perceived protective behavioral change was associated with age, worry (*β* = 0.294, *p* < 0.001), positive coping (*β* = 0.196, *p* < 0.001), sex (*β* = 0.127, *p* < 0.001), negative coping (*β* = −0.111, *p* = 0.001), and perceived severity (*β* = 0.106, *p* = 0.005). The overall model was significant [*F*_(7,790)_ = 29.925, *p* < 0.001], with worry being the most predictive variable of perceived protective behavioral change. Detailed results are shown in Table 4.

**Table 4:** Results of multiple linear regression on perceived protective behavioral change (*N* = 798).

Variable	*B*	*SE*	*β*	*t*	*p*	95% CI for *B*	
						Lower	Upper
Constant	30.149	2.830		10.652	<0.001	24.594	35.705
Age (ref: ≤30 years)							
31–45 years	2.134	0.716	0.103	2.981	0.003	0.729	3.540
≥46 years	3.522	0.926	0.134	3.804	<0.001	1.704	5.339
Worry	0.430	0.057	0.294	7.533	<0.001	0.318	0.542
Positive coping	0.360	0.059	0.196	6.113	<0.001	0.244	0.476
Sex	3.039	0.772	0.127	3.935	<0.001	1.523	4.556
Negative coping	−0.172	0.053	−0.111	−3.271	0.001	−0.275	−0.069
Perceived severity	1.219	0.429	0.106	2.844	0.005	0.378	2.060

Model: *F*_(7,790)_ = 29.925, *p* < 0.001; VIF: 1.032–1.522; SE, standard error; β, standardized coefficient; CI, confidence interval.

## Discussion

This study provides valuable insights into the perceived changes in protective behaviors among Chinese residents during the early stage following the lifting of 3 years of strict disease control and prevention measures. Given the distinct experience of COVID-19 prevention measures in China compared to many other countries, understanding these perceived changes is crucial for assessing the immediate impact of the policy shift. This study also identifies factors that either promote or impede the overall change, providing valuable insights for policymakers to design strategies that ensure public safety during transitions in public policies. Such knowledge is essential for preparing for future public health crises, as it highlights behavioral patterns that emerge with policy shifts and helps predict public responses to similar adjustments.

Generally, the results of this study indicate that self-imposed protective behaviors increased during this period, with all behaviors rising—some minimally and others substantially. The result indicates that, even after 3 years of strict prevention and control regulations, the public continued to perceive the need for ongoing preventative practices and actively engaged in managing their own and their families’ health. Notably, avoiding dining out or gathering with friends exhibited the greatest increase in scores, with most participants reporting a rise in this behavior. However, in accordance with previous studies (14), many people reported a decrease in maintaining a social distance of more than 1 m. The possible explanations for this observation are complex. First, social distancing is a mutual behavior requiring everyone’s participation. Over time, people may experience “behavioral fatigue,” leading to less consistent adherence (40). Once a person breaks the one-meter rule, the safe distance is compromised. In Chinese culture, the belief in “being strict with oneself and lenient with others” makes it unlikely that individuals would enforce social distancing after government policies are lifted. Moreover, research suggests that much of behavior change stems from indirect effects, where people mimic others (41). When policies are lifted, these indirect effects may weaken, further reducing adherence. Second, while people can choose to avoid gatherings for entertainment, such as parties or movies, some social activities are obligatory as society returns to normal after the lifting of the dynamic zero-COVID policy. For instance, schools have resumed classes and businesses have reopened. As offices, classrooms, public transportation, and streets become more crowded, these environmental changes make it impossible to maintain social distancing solely based on personal choice. Engaging in regular physical activity had the lowest mean score, indicating minimal change relative to pre-policy levels. This may be attributed to shifting priorities and the complexities associated with maintaining an exercise routine during this period. Additionally, our survey focused on the purchasing patterns of medical supplies and drugs, including masks, antiviral drugs, disinfectants, and antigen test kits. The substantial quantities of these items purchased underscore their critical role in personal and family protection. Ensuring adequate stock of these items is essential to meet ongoing demand and support public health efforts.

Regression analysis revealed that older and female individuals were more likely to exhibit more positive changes in protective behaviors. Similarly, Qin et al. (42) found that female and older college students tended to perform better at protective measures. Moran et al. (19) also showed that older people and women reported a high level of adherence to public health guidelines. This tendency might reflect a heightened sense of vulnerability and responsibility among these groups (43–45). Although demographic factors themselves are not modifiable, understanding their impact can help identify key groups that exhibit different levels of protective behaviors. Older individuals and females, who were more likely to show positive changes, may benefit from continued support to sustain these behaviors. In contrast, younger individuals and males may need additional targeted interventions to help improve their adherence to protective practices.

Worry was found to be the strongest predictor of perceived protective behavioral change, significantly influencing individuals to adopt protective actions. Alongside worry, perceived severity also showed a significant association with protective behaviors. Our results are in line with the findings of previous studies (33, 46) and reinforce concepts from theories such as HBM and PADM. These results emphasize the crucial role of emotional and cognitive factors, particularly worry, in motivating protective actions. While both worry and perceived severity are key drivers of protective behaviors, worry stands out as the most influential factor. However, excessive negative emotions and heightened risk perceptions may lead to psychological issues and increased public fear. Dryhurst et al. (16) noted that higher perceived severity of COVID-19 drive protective behaviors but can also increase stress and anxiety. Holman et al. discussed the impact of high worry levels on mental health and the importance of supportive interventions to manage stress while promoting protective behaviors (47). Rubin et al. (48) found that while worry can drive protective behaviors, it can also lead to significant public panic if not managed with clear and accurate information. To balance these effects, effective communication strategies should include providing accurate information, offering practical guidance, fostering resilience through positive messaging, emphasizing collective responsibility, and maintaining transparency and empathy. These approaches will help individuals manage worry and perceived severity while maintaining necessary protective behaviors (48–50).

Our findings suggest that positive coping was positively associated with the overall perceived change in protective behaviors, whereas negative coping was negatively associated with this change. Positive coping is defined as using constructive methods to solve problems and alleviate stress, thereby enhancing psychological resilience. In contrast, negative coping involves avoidant or ineffective strategies that may temporarily reduce stress but often lead to further psychological or behavioral issues (51). Positive coping strategies, such as problem-solving, seeking social support, and positive reframing, are linked to increased adoption of protective behaviors during a pandemic. These strategies foster a sense of control and resilience, leading individuals to adhere more to health guidelines like mask-wearing and social distancing (52). Research has shown that such adaptive coping mechanisms are associated with proactive health behaviors and reduced stress levels (53, 54). In contrast, negative coping strategies, such as avoidance, self-blame, and venting negative emotions, are associated with lower adherence to protective behaviors. These maladaptive strategies can increase stress and anxiety, leading to neglect of health guidelines and risky behaviors (47, 55). The psychological burden from these negative strategies reduces the capacity for individuals to engage in effective protective measures. Such findings provide empirical support for Lazarus and Folkman’s Stress and Coping Theory in addressing the role of coping responses in the face of adversity in behavior health (32).

### Implications

This study provides essential insights into public responses to the lifting of long-term disease control measures, particularly in terms of protective behaviors. First, the increase in behaviors such as avoiding gatherings, alongside the reduced adherence to social distancing, indicates that behavioral adjustments are shaped by a combination of external policies, cultural factors, and individual risk perceptions. This suggests that certain behaviors, like social distancing, may be more difficult to maintain without clear threats and mandatory requirements, while others—like avoiding gatherings—are more easily sustained. Second, understanding the multifaceted factors influencing perceived protective behavioral changes is crucial for developing targeted interventions and policies that sustain public health behaviors during and after crises. Policymakers can focus on demographic groups that are more likely to sustain positive behavioral changes, such as older individuals and women, while designing interventions that target younger individuals and men to improve their adherence to protective practices. Moreover, the role of emotional and cognitive factors, particularly worry and perceived severity, is crucial in shaping protective actions. Managing public worry through transparent and empathetic communication is essential to avoid excessive fear and stress while promoting necessary health behaviors. The dual role of worry and perceived severity in shaping protective behaviors, mental health, and public panic underscores the need for more nuanced models of emotional and cognitive responses in health behavior research. Additionally, relevant psychological support that promotes positive coping strategies and reduces negative coping is essential for managing stress constructively while maintaining adherence to protective behaviors. Such support can have lasting and profound impacts, enhancing resilience and preparedness for future adversities. By addressing emotional drivers and behavioral fatigue, public health strategies can better ensure long-term safety and preparedness during transitions in policy and crisis management.

### Limitations

Several limitations should be considered when interpreting these findings. First, given the cross-sectional and self-reported nature of this study, perceived behavioral change may not have represented actual behavioral change due to recall and social desirability bias. However, this study provides a tentative outline of the behavioral responses of the public during this particularly stressful situation. Future research could build on this by comparing perceived and observed behaviors to provide a more comprehensive picture. Second, the use of online surveys may exclude individuals who have limited internet access or lower digital literacy, potentially affecting the generalizability of the findings. Future studies should consider using multiple data collection methods to reach a more diverse audience. Third, the convenience and snowball sampling methods employed in this study could have introduced selection bias. These methods rely on participants referring others within their networks, which may lead to a sample that is not fully representative of the broader population. As a result, there may be an overrepresentation of certain demographic groups, such as females or individuals with higher education levels. Future research should consider employing more targeted recruitment strategies and systematically monitoring demographic diversity to ensure a more representative sample. Incorporating stratified sampling techniques or larger, randomized samples could help mitigate these biases. Lastly, a self-designed questionnaire based on previous research was used to ensure the timeliness of the survey. Although the Cronbach’s alpha coefficients were within an acceptable range, the reliability and validity of the questionnaire should be tested in future studies.

## Conclusion

This study can contribute to a better understanding of the behavioral responses of Chinese residents in the early stage following the lifting of the three-year dynamic zero-COVID policy. Our findings indicate that, during this period, Chinese residents generally reported increases in their self-imposed protective behaviors, with varying magnitudes across behaviors. To meet the ongoing public demand, public health practitioners and policymakers should ensure the adequate stockpiling of essential medical supplies, including masks, antiviral drugs, disinfectants, and antigen test kits. Tailored interventions are necessary to support younger individuals and males, who may require additional assistance to improve adherence to protective behaviors. The current study underscores the need for effective communication that provide accurate information, practical guidance, and foster collective responsibility to reduce excessive worry and perceived severity, thereby promoting protective behaviors. Psychological support that encourages positive coping and reduces negative coping is essential for managing stress and sustaining protective behaviors. Finally, this study highlights the need for future research to develop more comprehensive models in health behavior that balance public panic with safety measures, and to address the limitations by using longitudinal designs, incorporating diverse data collection methods, employing more representative sampling strategies, and further validating survey instruments to ensure reliability and generalizability. Overall, our findings offer valuable insights for designing interventions to support protective behaviors and enhance public health preparedness during policy transitions.

## Data Availability

The raw data supporting the conclusions of this article will be made available by the authors, without undue reservation.

## Footnotes

^1^ http://www.wjx.cn/
