# Supplementary material for: Perceived protective behavioral changes in Chinese residents post-dynamic zero-COVID policy lifting: a cross-sectional study
Source: Front Public Health. 2024 Oct 30;12:1439749. doi: 10.3389/fpubh.2024.1439749 (PMC11557542; doi:10.3389/fpubh.2024.1439749)
Supplement: Supplementary file 1 [file Data_Sheet_1.docx]

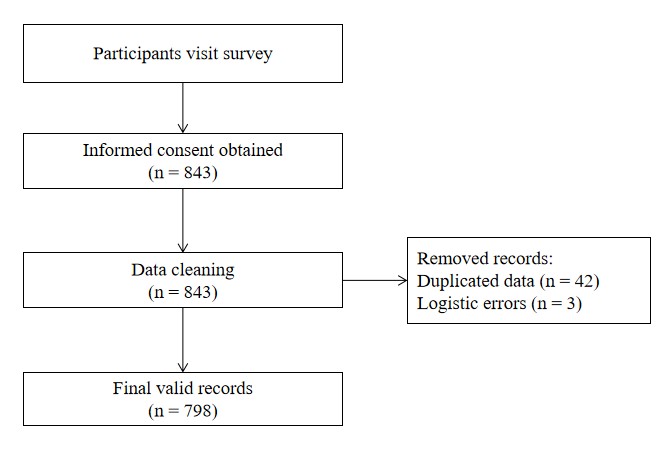


**sFigure 1** Data Cleaning Flowchart

**sTable 1** The summary of the questionnaires used in the study

| Questionnaires | Description | Number of Items | Sample Item | Source/Reference |
| --- | --- | --- | --- | --- |
| Basic information | Gathers basic information | 20 | "What is your age?" | Developed for this study |
| COVID-19 related perceptions | Assesses participants' perceived susceptibility to COVID-19, perceived severity, perceived impact of COVID-19, fear, and worry | Perceived susceptibility to COVID-19: 2  Perceived severity: 1  Perceived impact of COVID-19: 4 | “Please rate the likelihood of contracting COVID-19” | Adapted from Mo et al. (2021) |
| Negative emotions | Measures fear and worry | Fear: 1  Worry: 8 | “Please rate your level of fear of COVID-19” | Adapted from Mo et al. (2021) |
| Coping style (Trait Coping Style Questionnaire, TCSQ) | Evaluates participants’ style of coping with life events, including positive and negative coping | 20 | "You are able to quickly forget unpleasant experiences." | Jiang (2021) |
| Protective behavioral change | Assesses participants’ protective behavioral change since the release of “the 10-point measures” | 17 | “You avoid dining out or gathering with friends” | Adapted from Yang et al. (2021), Yıldırım and Güler (2022), and Yuan et al. (2022) |

**References:**

Jiang Q. Trait coping style questionnaire (in Chinese). Chin J Behav Med Sci. 2001;10:36-7.

Mo PKH, Fong VWI, Song B, Di J, Wang Q, Wang L. Association of Perceived Threat, Negative Emotions, and Self-Efficacy With Mental Health and Personal Protective Behavior Among Chinese Pregnant Women During the COVID-19 Pandemic: Cross-sectional Survey Study. J Med Internet Res. 2021;23(4):e24053.

Yang K, Liu H, Ma L, et al. Knowledge, attitude and practice of residents in the prevention and control of COVID-19: An online questionnaire survey. J Adv Nurs. 2021;77(4):1839-55.

Yıldırım M, Güler A. COVID-19 severity, self-efficacy, knowledge, preventive behaviors, and mental health in Turkey. Death Stud. 2022;46(4):979-86.

Yuan T, Li XD, Zhang M, Tao XB, Xu SJ, Liu H. Impact of the eHealth literacy, knowledge and attitudes on COVID-19 prevention behavior among residents in the second year of the COVID-19 pandemic: A cross-sectional study in Anhui Province, China. Front Public Health. 2022;10:1015803.
